# The experiences and challenges faced by rehabilitation community service therapists within the South African Primary Healthcare health system

**DOI:** 10.4102/ajod.v6i0.311

**Published:** 2017-09-26

**Authors:** Lieketseng Ned, Lizahn Cloete, Gubela Mji

**Affiliations:** 1 Centre for Rehabilitation Studies, Department of Interdisciplinary Health Sciences, Faculty of Medicine and Health Sciences, Stellenbosch University, South Africa; 2 Division of Occupational Therapy, Department of Interdisciplinary Health Sciences, Faculty of Medicine and Health Sciences, University of Stellenbosch, South Africa

## Abstract

**Background:**

Twenty-two years after the promulgation of a plethora of progressive health policies since 1994, the South African public health system reflects a number of stumbling blocks regarding implementation. Rehabilitation professionals are not sufficiently equipped nor allowed the opportunity to comprehensively implement Primary Healthcare (PHC) from a bottom-up approach, thus engaging communities. Training on addressing social health determinants and their impact on ill-health and health outcomes is inadequate. The inadequate understanding of the advocacy role that rehabilitation professionals could play in addressing social health determinants remains a challenge in healthcare. Rehabilitation, a pillar of PHC, remains poorly understood in terms of its role within the health system.

**Aim:**

We argue for rehabilitation as a vehicle for addressing social determinants of health with community service practitioners playing a critical role in addressing the inequities within the healthcare package.

**Setting:**

The article reflects the opportunities and challenges faced by rehabilitation community service therapists in the delivery of rehabilitation services in a rural area of the Eastern Cape province of South Africa.

**Methods:**

A single case study from the perspective of a researcher was used to explore the experience and reflection of the first author during her community service as an occupational therapist.

**Results:**

The case study highlights some existing gaps within the delivery of rehabilitation services in the rural Eastern Cape. A community service package with a specific approach towards addressing social determinants of health for persons with disability at a community level is suggested.

**Conclusion:**

Advocating for a rehabilitation service package to shift to community-based levels is critical. It is envisaged that a community-based approach will facilitate an understanding of the barriers faced by persons with disabilities as constituting disability, thus facilitating learning about the disabling consequences of the rural environment coupled with the system as experienced by persons with disabilities.

## Introduction

In 1998, the South African Department of Health (DOH), as a response to challenges associated with implementation of Primary Healthcare (PHC), created a one-year period of community service, with focus on underserved, primarily rural, areas (DOH 2000a). Health professionals, initially doctors, dentists and pharmacists, were required to do community service on completion of their training. A further seven professional groups followed in 2003, including physiotherapists, occupational therapists, speech therapists, clinical psychologists, dieticians, radiographers and environmental health officers (DOH 2000b). Placing new graduates for community service has been (and still is) largely overshadowed by the lack of both human resources to supervise these new graduates and financial resources to enable efficient and effective delivery of appropriate services (Hatcher et al. 2014). The gap between the newly qualified graduates’ skills set, the reality of service needs, context of service users and available resources of their services is a well-documented challenge (Reid 2001).

As the DOH (2000, 2007) noted:

‘the main objective of Community Service internship by health professionals was to ensure improved provision of health services to all citizens of our country. In the process, this also provided our young professionals with an opportunity to develop skills, acquire knowledge, behaviour patterns and critical thinking that will help them in their professional development.’ (p. 1)

With reference to district health institutions in particular, it was clear how this goal was aimed at bringing more services to the people and address insufficient human resources (Reid 2001). However, South Africa still faces shortages of human resources, especially within the public sector and in rural areas (DOH 2011a). This shortage poses a challenge to the implementation of many health policies including the National Health Insurance (NHI) and undermines equity (Doherty & Couper 2016). Hendricks et al. (2015) caution that inequities in health could be further exacerbated if the decentralisation proposed by the NHI gets poorly implemented. Graduates placed in rural areas face inequities in health system and rehabilitation professionals such as occupational therapists and physiotherapists are often supervised by other professionals who have limited or no training in dealing with disability and rehabilitation (Hatcher et al. 2014; Reid & Conco 1999). As the placement of more professionals is happening, new community-based training and service package models are required to equip graduates to serve in rural areas, be responsive to the needs and able to function in less resourced contexts (Doherty & Couper 2016; WHO 2010a). The process of creating a health system that is responsive to both disability and rehabilitation needs of people with physical, sensory, psychosocial and learning impairments is inevitably compromised by mediating differences in opinions for the identification of focus areas for service provision and resource allocation. Professional development and supervision are crucial in order to build practitioner skills, equitably distribute and retain professionals in rural underserved areas (Hatcher et al. 2014).

Worldwide, poverty and health disparities exist and are increasing (Thomas 2014). Poverty does not only exclude people from the healthcare systems’ health benefits but also restricts them from participating in decisions that affect their health (United Nations 2013). Despite some positive changes alluded to in the 2015 *South African Health Review*, South Africa’s health outcomes remain below what is expected from the current health expenditure. There are existing gaps between policy imperatives and implementation of the healthcare system, and policy implementation is inefficient (Mayosi et al. 2012; Naledi, Barron & Schneider 2011). Some of the reasons for the healthcare system’s ineffectiveness include (but are not limited to) inadequate implementation of PHC and lack of contextual intersectoral action for social health determinants and indicators. Healthcare workers are not sufficiently equipped to implement PHC and have too little training on how to address the social health determinants of health and wellness. The advocacy role and actions that health and rehabilitation professionals could play to address social health determinants remain unrecognised within the healthcare system. For example, Weeramanthri and Bailie (2015) propose that the health status of communities could be addressed by providing basic needs of communities such as improvements in housing, sanitation, drinking water, education, employment, working conditions, food supply, transport infrastructure and other social health determinants. Investing in these basic supports, as Naledi et al. (2011) suggest, is critical in improving health outcomes. Yet, such interventions remain poorly implemented within the current South African health system, resulting in a significant negative impact on the health sector (Naledi et al. 2011).

The reality of South Africa is that the health system still operates in a context where the above-mentioned basic needs for health are still not present in many communities. This is especially relevant for a number of rural communities in the different provinces of South Africa. The dominating focus of the health system on incidence and prevalence of diseases underestimates the importance of disease prevention, especially in resource-limited communities (Dookie & Singh 2012). Naledi et al. (2011), for example, recount that different programmes within the national and local Departments of Health are working in parallel, particularly health promotion programmes which are often seen as the marginal players alongside bigger programmes such as HIV and TB. They also recount that in-service training is weak and often provided in programme-specific silos instead of holistically. These demonstrate a reductionist approach with its other limitations including limited interprofessional exposure, inadequate orientation in understanding the role of social determinants on health outcomes (Diez Roux 2012) and poor understanding of the advocacy role health and rehabilitation professionals could play in addressing social health determinants and limited capacity to effectively advocate (Ng et al. 2015). The implication of the above-mentioned factors is a weak health system with professionals who continue to lean more towards an institution-based medical model and disease-specific ways of addressing ill-health, thereby failing to provide client-centred services that are contextually specific with focus on health promotion and disease prevention. Reductionist approaches furthermore tend to perpetuate the notion of clients being passive receivers of services rather than proactively participating in the prevention of ill-health and promotion of health within their communities (Naledi et al. 2011).

Reductionism may perpetuate a health system that is reliant on expert-driven and standardised approaches, which are central to biomedicine within the health system and hardly address the challenges of social health determinants that clients face at community level. Hence, it is argued that South Africa has failed to establish a strong district health system (DHS) and, particularly, to develop and manage human resource capacity at the district level (Mayosi et al. 2012; Naledi et al. 2011), which would connect with populations. During policy implementation, there is still no clear integrated plan in place for human resource support that will deal with health-related basic support challenges such as water, sanitation and food security (as main determinants of health and wellness) at community level. Despite the efforts to manage human resources for health (DOH 2011b), the resource allocation for implementation remains inadequate. Provincial governments still control much of the financial resources and maintain decision making power. In addition, the institution-based medical model still dominates, making it difficult to act innovatively and implement new strategies that create bridges between health institutions and the communities they serve (Dookie & Singh 2012; World Health Organization 2007). Infrastructure challenges in rural areas exacerbate these challenges, especially for the rehabilitation health service user (Gaede & Versteeg 2011; Matsoso & Strachan 2011; Versteeg, Du Toit & Couper 2013), with regard to achieving their rehabilitation goals. If the infrastructure at community level is not conducive, this can act as a barrier to health, wellness and community participation. This article uses first-hand experience contextualised by literature to highlight critical service issues and to advocate for rehabilitation as a vehicle for strengthening the health system to address social determinants of health. In this case, community service practitioners are positioned as critical human resources to potentially fill the highlighted gaps.

## How South Africa implemented Primary Healthcare

The 1994 political changes that moved South Africa from an apartheid era to a democratic elected government brought a plethora of policies that were developed with the aim of bringing change in the lives of South Africans, particularly vulnerable groups such as persons with disabilities in underserved and rural environments. The major focus of the change included developing a healthcare delivery system that is based on a decentralised PHC district system. Some of the fundamental improvements related to the health arena included an increase in basic infrastructure (which included housing, water and sanitation) as well as the implementation of the strategies for poverty alleviation (Mji 2012). The 1997 White Paper for the Transformation of the Health System ushered us into an era of health service transformation (DOH 1997). The principles that were underpinned by the Alma Ata Declaration were conceptualised and plans proposed for implementation by 2000. The shift was structurally focused on building and upgrading clinics as well as the overall establishment of the DHS. This comprehensive PHC as envisioned at Alma Ata explicitly outlined a strategy that would respond equitably, appropriately and effectively to basic health needs and also address the underlying social, economic and political causes of poor health (County of Los Angeles Public Health 2013; Magnussen, Ehiri & Jolly 2004). Successful implementation of PHC thus required a change in socioeconomic status, distribution of resources and a focus on responsive health systems with emphasis on basic health services. The approach was underpinned by all the factors key to the success of a public healthcare system such as universal accessibility, emphasis on disease prevention, health promotion, community participation, self-reliance, rehabilitation and intersectoral collaboration (Dookie & Singh 2012; Magnussen et al. 2004). The DHS, as vehicle for PHC, was formalised in 2003 (Republic of South Africa 2005). Although PHC, as social justice policy in itself was combined with other legislative policy and resource allocation measures, it has not been enough to meet transformation targets for the improvement of population health (Rispel 2016).

Health systems’ activities and their outcomes are better obtained when people’s basic needs are met, first and foremost. A prerequisite for meeting basic health needs is that a health system’s infrastructure allows people to guide the process of responding to their health needs according to their context (Matsoso & Strachan 2011; World Health Organization 2010a). That was the health vision of the 1994 newly elected democratic government of South Africa. It appeared that this vision was being driven by a deeper understanding that health is life (Mji 2012) and any tangible success of the newly elected government stood on the premise that the area of health should be given priority. Realistically, instead of implementing this comprehensive PHC strategy of 1978, it appears that healthcare service provision focused mainly on providing selective primary care, instead of providing an integrated PHC service that addresses social health determinants (Dookie & Singh 2012).

## Challenges with implementation of Primary Healthcare in South Africa

Considering the PHC philosophy’s potential to contribute to improved community health, challenges exist relating to effective community participation, intersectoral collaboration and optimal use of available resources (Dookie & Singh 2012; Morgan & Ziglio 2007; Naledi et al. 2011). Such available resources include indigenous health knowledge that is lying dormant at the community level especially in rural areas. Mji (2012) asserts that as much as the new government’s health agenda was geared towards shifting both human and financial resources from the large incumbent tertiary institutions to the district level, initially, there remained no clear plan regarding the percentage of shifts. This unclear plan could explain the inequities still experienced in human resources for health with rural areas being disadvantaged the most (Hatcher et al. 2014). It was not clear how these sectors would relate to each other or to referral systems. When referral happens, there are no human resources to pick up these referrals at the community level. The most challenging area that was neglected at these service points of health delivery was health promotion and disease prevention information for both primary and secondary illness. Provision of a limited range of PHC services thus could perpetuate the revolving door syndrome for health conditions that could be prevented in the first place (Mlenzana & Mji 2010).

The situation at the Community Healthcare level is difficult because of a shortage of human resources, resource allocation problems and the type of model used to deliver PHC. Overcrowding at these facilities is the order of the day (ANC 1994; Cook 2005; Gessler, Msuya & Nkunya 1995; Mash 2004; Mlenzana & Mji 2010; Zonke 2005). The lack of proper planning on implementation of PHC by the South African government resulted in a PHC system that is still burdened with:

- Absorbing most of the budget but failing to address inequities in health at ground level and a PHC system that is predominantly used by the poor who remain sick as inequities persist (Mji 2012).
- Fragmentation, with little attempt at an integrated, interdisciplinary approach that links PHC to health promotion and disease prevention (Dookie & Singh 2012; Werner & Sanders 1997).
- Poor or inappropriate patient education and advice by healthcare providers and no referral to secondary levels of healthcare provision for patients with complex health conditions (Mlenzana & Mji 2010).
- Lack of interprofessional research initiatives (Mji 2012) and evidence on local PHC models that have worked, or the moving of evidence to action. In Africa specifically, much of the research has been concentrated in English-speaking countries.
- Lack of commitment to infrastructure and human resources for the implementation of rehabilitation, thus denying health promotion, disease prevention and participation, which would facilitate wellness and quality of life, all critical elements of PHC (Mji 2012).
- Unspent budgets because of poor planning and a lack of human resources. This exacerbates health inequalities and inadequate service delivery (Mlenzana & Mji 2010).

The above-mentioned problems were a result of the insufficient attention given to disease prevention, health promotion and community participation as part of the implementation of PHC (Dookie & Singh 2012; Hess-April 2013; Mji 2012; Sherry 2015). Following these failures, PHC re-engineering which emphasised community-based services by reaching out to households (DOH 2010) and NHI (DOH 2011a) were introduced. In response to these developments, the Western Cape DOH (2013) developed the Healthcare 2030 draft, which also poorly defined rehabilitation services. Part of the challenge is that none of these above-mentioned policies developed a service package for rehabilitation services (Hess-April 2013). The shortcoming was then acknowledged and a national task team was constituted to formulate a rehabilitation service delivery strategy within the PHC framework. To date, the Framework and Strategy for Disability and Rehabilitation (FSDRSA) (DOH 2015) was developed. It is left to be seen how it will improve the challenges of implementing PHC.

## The current rehabilitation status in South Africa

Rehabilitation is a pillar of PHC and has as its primary goal the integration of persons with disabilities within their environments. Sherry (2015) notes that rehabilitation remains excluded and poorly understood in healthcare. As a component of healthcare, access to rehabilitation is limited in both poorly resourced and well-resourced provinces (with rural areas being far worse) with human resources for the provision of these services being subject to challenges, especially in the public sector (Sherry 2015).

The National Rehabilitation Policy suggested that rehabilitation services must be delivered as part of PHC according to Community-Based Rehabilitation (CBR) principles (DOH 2000, 2010). CBR shared common principles with PHC; however, the challenge has been the limited understanding that CBR not only refers to services provided outside of institutions but also refers to a general philosophy which aims at the inclusion and full participation of people with disabilities in all aspects of community life (Sherry 2015). The existing FSDRSA appears to reflect this understanding (DOH 2015). It is then critical for issues raised in this paper to be noted as provinces are tasked to develop the implementation plan for the FSDRSA (DOH 2015). The PHC approach, on which healthcare service delivery is based, highlights the need for these services to be comprehensive and transformative at the community level (DOH 2013), regarding community members as active participants in determining steps that will influence their health (Sherry 2016). A PHC approach challenges societies to identify and address the causes of poor health in their communities, make provision for basic health needs and encourage communities to become empowered (Dookie & Singh 2012; Sherry 2016). Although rehabilitation is considered as one of the components of PHC, it is rarely included in PHC programmes (Mpofu 1995; Sherry 2015). The PHC provision continues to be fragmented and uncoordinated with some rehabilitation services less accessible to some sections of society than to others.

The quest to address inequities and improve health reveals a tendency to focus on identifying the problems and needs of populations who require professional resources, rather than on the assets that already exist within these populations. These neglected assets include the health-related indigenous knowledge (IK) that is lying dormant in communities. This deficit perspective creates a high level of dependency on hospital and welfare services (Morgan & Ziglio 2007). It (deficit approach) also differs substantially from an IK system way of addressing ill health, in which healers are merely facilitators, with healing being an interactive experience for clients, their families and the healer (Moshabela, Zuma & Gaede 2016). In this system, the family plays an active participatory role in tracing the start of illness including the events that might have contributed. In this regard, for healthcare to not be conversant with the cultural ways of the community seems problematic and divides healthcare, leaving critical components such as rehabilitation on the margins.

Indigenous knowledge is a valuable resource, but has historically not been deployed for health gain in South Africa and elsewhere in the world. Health-related scientific knowledge has been and still is being prioritised over other forms of knowledge like IK, resulting in a hierarchical classification of knowledge (Moshabela et al. 2016). In this case, IK is practised in secrecy in indigenous communities and is thus lost to society. For example, Boneham and Sixsmith (2005) found, in a northern town in the UK, that the voices of indigenous older women were rarely heard in debates about health. Similarly, in South Africa, a study by Mji (2012) suggested the need for understanding health from the perspective of the users, especially the older members of indigenous communities as they most often give advice when a family member is ill. This study demonstrated the tensions, mistrust and conflict between the health system and the community as indigenous communities felt that the hospitals had brought ill health to their communities by not focusing on what they perceive as critical indicators of health, which are social health determinants.

The WHO definition of health states that health is not merely the absence of disease or infirmity but a state of complete physical, mental and social well-being. In Mji’s (2012) study, the older community members criticised this definition by highlighting the absence of critical aspects of health for their communities in this definition. Mji (2012) shared a view of health, as described by older Xhosa women in Madwaleni (Eastern Cape), which includes (amongst other things) being able to participate in the key activities and functions of their villages, being able to produce food for the village as well as bringing up children from childhood to adulthood. When these social health determinants are incorporated into healthcare, they are effective at the level of disease prevention and health promotion (Dookie & Singh 2012; Sherry 2015). Though a promise was made of officially integrating traditional healers into the NHI landscape following the appointment of an Interim Traditional Health Practitioners Council in Pretoria on 12 February 2013, there is a glaring absence of both indigenous approaches to healthcare and indigenous healers in the White Paper on the NHI. This raises questions with regard to whether such community-specific definitions of IK systems (Moshabela et al. 2016) would ever be recognised for the critical contribution and role they could play in the implementation of re-engineering of PHC, the new health plans on NHI and the implementation of the FSDRSA for policy aspirations to be realised at ground level. This is particularly critical as various studies still indicate challenges and tensions related to poor recognition of the indigenous health system (Mji 2012; Moshabela et al. 2016). Its recognition remains symbolic on policy.

The above indigenous understanding of health by Mji (2012) reveals the notion of health as an active process of participation is often not made explicit by rehabilitation professionals. This definition strongly reinforces rehabilitation as a critical aspect of health. The different conceptualisations of what it means to be healthy within biomedicine and indigenous perspectives (Moshabela et al. 2016) invite debate to clarify possible disjunctures in service planning and service provision. The neglect of the indigenous perspective explains Sherry’s assertion that rehabilitation continues to be excluded and poorly understood within healthcare (2015). The above indigenous perspective on health has the potential to fill the gap as it situates rehabilitation within public healthcare. At PHC level, appropriate and relevant rehabilitation services will facilitate engagement in functional activities that will enhance the quality of life and wellness of community members. We further posit rehabilitation as a necessary imperative for those who already have disabilities to assist with secondary prevention and facilitate their active engagement and participation, which will lead to successful community integration. This successful integration can only happen when cultural and contextual issues of that population are taken into consideration.

Below is a case study illustrating challenges within a health system as experienced by a graduate during her year of community service practice. A case study is very useful when seeking to explain the how and the why of social phenomena in context (Yin 2014). Case study, as a methodological design arises from a desire to understand complex social issues by allowing investigators to focus on a ‘case’ and retain a holistic and real-world view perspective. This method was appropriate to interrogate the complex issues brought forward in this paper.

## A case study of community service and related systemic challenges within the health system (a reflection of the first author’s experience)

In 2010, the first author was placed in a rural area for her Occupational Therapy community service year in the Eastern Cape province of South Africa. This area was and still is an under-resourced, rural context where geographical location is a huge barrier to accessing general services. The community is characterised by poor infrastructure and scattered villages in which the majority of households do not have access to potable water, electricity or proper sanitation. Public transport is limited and the sub-standard gravel roads make travelling difficult, long and sometimes impossible when the weather is bad. When people travel to town in the morning or when they are referred by the clinic to the district hospital, they return late in the afternoon, leaving the home, its activities and often children unattended or watched by a neighbour.

Primary care clinics were available to surrounding villages and managed by nurses. No rehabilitation therapists were designated to these clinics because rehabilitation services are not currently included in the policies regulating re-engineered district health teams (Pillay & Barron 2011). Many adults and children with physical, sensory and psychosocial disabilities were confined to their homes with no access to rehabilitation services, this despite the community service policy objective of ensuring accessible and equitable comprehensive healthcare. The majority of referrals for rehabilitation at the hospital were for patients with neurological conditions such as strokes in adults and cerebral palsy in children. The clinic statistics indicated health problems typical of a rural context with deep-rooted poverty and a high prevalence of major health issues, that is, TB, HIV or AIDS, high teenage pregnancy and chronic diseases of lifestyle (mainly hypertension, arthritis, diabetes and mental health disorders including substance-induced psychosis). A high unemployment rate was evident with many people sustaining their livelihood through selling products on the streets while others had left the community in search of work in the cities.

The goal of our work was the provision of rehabilitation programmes that focused on health promotion, education and prevention and early identification at community level. It soon became evident that achieving this goal was challenging as it required skills that I was not sufficiently taught during my undergraduate training, particularly advocacy skills. I often felt ill-prepared for the task at hand. We had to be strategic about accessing transport and collaborated with other sectors like social development and the school nurses to access lifts from their transport. We became familiar with the other services provided by other sectors and collaborated in terms of referrals and case discussions. We received invitations to schools and clinics for health promotion and health education events. Our work at community level, initiated by requests from the school nurses, was often disregarded by hospital management, despite the fact that it gave an increasing number of patients’ access to rehabilitation and care. Our usual working day at the hospital involved sitting and waiting for clients. The aim to raise awareness of the availability of rehabilitation services and thus the health promotion and disease prevention benefits thereof could not be achieved. We felt that even within the institution, rehabilitation was dismissed by hospital management, nurses and doctors, with no prioritisation of resources for rehabilitation. This was exemplified by the refusal of the nurses and doctors to attend a seminar, which would have introduced them to our rehabilitation service, work out a referral system and how we could work more efficiently together as a team instead of silos. It was also demonstrated by limited or no budget allocation. They would say ‘we prioritise essential services (read medical services)’. This reflected a disregard for collaborative practice and highlighted challenges relating to professional superiority versus mutual respect. The lack of interest on a basic principle of teamwork undermined interdisciplinary and collaborative practice, referral pathways and continuum of care and resulted in poor utilisation of already limited resources. Furthermore, service users are not aware of the services available to them and how they can access them. There is no community participation in the design of these services, and often, some of the accessibility barriers are the already mentioned ineffective systemic and structural issues.

## Discussion

There is no doubt that implementing a one-year community service programme for newly graduated health professionals does significantly improve the availability of human resources within the public health sector system. However, the ineffective structures and systems to support this community service initiative fail to yield an impact on the opportunities available to reach individuals in need of rehabilitation services. The inadequate impact may in turn compromise health outcomes of communities. Institutionalisation, ineffective management, inaccessibility, lack of transport for professionals and clients and inequity in distribution of human and material resources continue to be barriers to the delivery of services at PHC level by compulsory community service providers including medical doctors, dentists, speech language-and-hearing therapy professionals and dieticians in South Africa (Khan, Knight & Esterhuizen 2009; Mostert-Wentzel, Frantz & Van Rooijen 2013; Paterson, Green & Maunder 2007; Penn, Mupawose & Stein 2009; Ramklass 2009; Reid 2001; Reid & Conco 1999). Some of the implications presented by this case study include services that continue to be inaccessible while rehabilitation professionals wait in their consultation rooms in the institutions for persons with disabilities to come to them for services. These clients remain unaware of the services available to them (Mji 2012; Ned 2013) and when they receive these, they often feel patronised by inappropriate treatment that do not address the social determinants of health (Mostert-Wentzel et al. 2013). Additionally, institution-bound providers may choose to leave the public sector or even the country because of low morale (Mostert-Wentzel et al. 2013). A single interpretive case study (Hess-April 2013) on Occupational Therapy community service providers in under-resourced rural communities revealed these several challenges as posed by the settings of practice. The dominance of the medical model (we see this in the imposed institutionalised services approach instead of providing CBR) and lack of resources to a system of bureaucracy remain the biggest encountered challenges documented (Hess-April 2013). Hess-April (2013) also found that the health system was not ready to accommodate the community service providers’ practice, though they had been equipped to provide services as guided by the local needs thus resulting in hegemony in practice. As shown in the reflective case presented and that of Hess-April (2013), this hegemonic encounter makes the community service providers display attitudes of defeatism, feelings of guilt, despondency and powerlessness. The skills to respond to power dynamics and interact with people in positions of power appear to be lacking (Hess-April 2013). It is recognised that the system needs to change but rehabilitation professionals also need to consider how they could develop and implement actions to address the system in order for their practice to become more facilitative of change. Skills such as advocacy, how to facilitate collaboration, negotiation, ability to influence decision making and conflict resolution could make a difference to the experienced situation (Hess-April 2013).

The content covered by undergraduate curricula in the health sciences limit students’ appreciation of health and how to address the underlying determinants of health. The dominating institutionalisation of services also hinders the providers from understanding these communities of practice. As a result, rehabilitation professionals fail to understand the health-related issues in the communities they are providing a service to. Competence to respond to cultural and diverse contextual demands is fostered when professionals engage at community level (Wentzel, Frantz & Van Rooijen 2013). Hess-April (2013) concludes that for community service providers to impact the contexts in which they practice, their education must ensure the development of competence to deal with the complexities of community service practice. This implies framing their preparation not just as learning but as a process of critical reflexivity that equips them to respond to power dynamics and intervene in matters as active agents of change.

Early discharge from tertiary and secondary levels of care and referral to primary level of care is still a problem experienced with no follow-up at community level, thereby impacting negatively on the community integration of persons with disabilities post-discharge as described by Zonke (2005) more than 10 years back. As per the DHS, part of the role of district hospitals was (is) to give support to rehabilitation services in the clinics and community, conduct disability awareness programmes and support the reintegration of persons with disabilities into the community (DOH 2002). However, insufficient attention had been given to the implementation of the PHC approach, which includes taking comprehensive services to communities with emphasis on disease prevention, health promotion and community participation. Hence, the later focus for PHC re-engineering aimed at the delivery of such services (DOH 2010) while the introduction of NHI aimed to ensure universal health coverage (DOH 2011a). In response to these developments, the Western Cape DOH (2013) developed the Healthcare 2030 draft, which also poorly defined rehabilitation services. Part of the challenge is that none of these above-mentioned policies developed a service package for rehabilitation services (Hess-April 2013) while South Africa is said to suffer from a quadruple burden of disease: maternal and child mortality, HIV and TB, non-communicable diseases and violence and injury (Pillay & Barron 2011). Rehabilitation indeed remains to be excluded and poorly understood within healthcare as described by Sherry (2015).

Furthermore, planned intervention programmes are not responsive and aligned to the actual needs of the community, resulting in poor continuum of care and secondary impairments. The result of this is a continued cycle of disease, ill health and disabilities in communities.

### The disjuncture between training and health system

The experience narrated in this case study demonstrates that the operational systems within the facilities did not provide opportunities for newly graduated professionals to offer their rehabilitation skills. Hence, in situations that required contextually relevant problem-solving, these rehabilitation professional felt ill-prepared. A consistent, strong advocacy strategy for CBR services is required at different levels in the hospital to ensure buy-in from management. A study on community service physiotherapists reveals that the compulsory community service provides opportunities for comprehensive care in a variety of settings, from homes, clinics and schools to hospitals (Mostert-Wentzel et al. 2013). Despite this evidence, the majority of community service therapists are still systemically and structurally forced to offer institution-based services. As such, they are not able to provide comprehensive contextually relevant rehabilitation at community level. Mostert-Wentzel et al. (2013) posited the absence of service learning in authentic diverse contexts as a barrier that hinders professional development and cultural competence. Furthermore, advocacy and community mobilisation components within rehabilitation remain underdeveloped. Similarly, new graduates doing community service often experience the disjuncture between their training received and what the system allows for (Frenk et al. 2010; Hess-April 2013). Thus, there is a need to facilitate alignment between higher education curricula and the operationalisation of health policies such as the FSDRSA (DOH 2015). Comprehensive PHC should not be limited in theory but emphasis needs to be placed on upskilling graduates to meet the service needs of rural communities while the health system allows room for such to be provided.

### Inadequate policy literacy

There is also the issue of inadequate policy literacy amongst rehabilitation professionals, health service users as well as the managers of community service professionals (Dube 2006; Duncan et al. 2011; Meyiwa 2010; Sherry 2011). Based on the first author’s experience, it seemed that managers were not aware of their obligation in terms of policies as well as the obligation of the staff they are supervising, leading to a lack of responsibility for implementation of current policies by rehabilitation professionals, especially in systems where red tape hampers service delivery. Similarly, Rispel (2016) refers to what Reid and Conco (1999) found as general management deficiencies in the public health system, unsatisfactory conditions of service and resource constraints as barriers to providing efficient and effective health and rehabilitation services.

### Strengthening the health system through focusing on social health determinants

The health system needs to start taking its rightful position by acknowledging the critical role of the social determinants of health in determining health outcomes and addressing equity issues in health. The health system should play a key role as an umbrella body driving intersectoral collaborations as one of its successes in achieving good health outcomes. This focus on social determinants drive relies heavily on partnerships with other sectors and communities to draw its indicators from the people it serves. The community service therapists could address these issues at community level, receiving and following up on clients to facilitate their integration. At community level, community service therapists would act as generalists intervening on a wide spectrum of issues that reflect the country’s quadruple burden of disease and disability (Mostert-Wentzel et al. 2013). In line with the national policies such as the re-engineering PHC and the FSDRSA (DOH 2015), addressing the burden of disease and disability includes addressing the social determinants of ill-health through prioritisation of disease prevention, health education and promotion. This implies that, in community service, the holistic rehabilitation team would address broader issues – specifically poverty. Thus, more opportunities to work with other sectors as demonstrated by the CBR (WHO 2010b) as a community development strategy to understand and address the underlying determinants of health as well as equity issues. It is worth noting that the CBR has been included in the current FSDRSA (DOH 2015). The community service therapists would play more of a role in facilitating, mediating between the community and sectors as well as assisting with advocacy work at community level. Though rehabilitation professionals may feel that this falls outside their scope of practice, Mostert-Wentzel et al. (2013) argue that rehabilitation could play a significant role doing advocacy work and driving intersectoral collaboration that could improve referrals to the relevant people who may address some of these social determinants. Often in these rural areas, other professionals and communities are unaware of the rehabilitation professionals and their services. Rehabilitation professionals can facilitate community participation in food production as a way to address the poverty issues. These social determinants of health must be addressed as well in order to fully achieve issues related to access, equity, affordability and good quality of care within our current health system. The indicators drawn from the community could then be used to draw up intervention plans, inform policy development and identify possibilities for research needed.

## Acknowledging the existence of multiple options of healthcare

In critically analysing the institution-based healthcare system and the role of patients as passive recipients of healthcare services, the healthcare system perpetuates dependency in the expert-driven and standardised approach it follows without building on what communities have and how they have been addressing health in the absence of a health practitioner (Flint 2015; Moshabela et al. 2016). Unlike biomedicine, indigenous ways of health are often communal with populations being actively engaged in the processes of promoting their health, and interventions are often tailored to the requirements of individuals and their families (Flint 2015; Mji 2012). Hence Mji (2012) calls for community engagement, which could be facilitated by these community service therapists in order to draw health and rehabilitation indicators, addressing health in a culturally, contextually relevant and equitable manner. This is particularly critical because of the existence of multiple options available to healthcare users in South Africa.

Yet, existing healthcare systems do not collaborate because of conflicting paradigms. The conflict of values and interests, mistrust and tension has brought ill health to many communities by disturbing the harmony of communities and their own way of living and managing their health (Mji 2012). The focus on ‘fixing’ health problems leads to communities wanting and lacking, thus the degeneration of structures and support already existing in communities. If the community-based health systems were non-existent all along, the question would be how were people surviving? Indigenous healthcare recognises the significance of the person’s personal experiences in ill health; ill health has its origins in the spirit world, for example one may be unhappy, not at peace, facing conflict in the home because of broken relationships, fear, etc. In contrast, biomedicine has no explanation for this aspect of illness and appears to see diseases as randomly contracted (Flint & Payne 2013; Mji 2012). Thus, social determinants of illness are marginalised by biomedicine, whereas they are perceived as causal factors for disease in these indigenous communities. Indigenous people are calling for a health system that does not separate them from their family and community in the intervention processes, and they are asking that their body, mind and spirit not be separated. We argue that the dominant healthcare system is still limited in terms of addressing the spiritual component of populations, thus threatening the future of healthcare (Mji 2012; Moshabela et al. 2016; Wreford 2005).

The health system cannot afford to regard diseases in isolation, but needs to look at the well-being of communities and at broader health indicators of participation, food production and the grooming of children (Mji 2012) as we navigate the re-engineering of PHC, introduction of NHI and implementation of the FSDRSA. These broader health indicators are what community service professionals could tap into at community level and bridge the widening gap between populations and rehabilitation services. While the intention of PHC as implemented in South Africa was to improve the health of public health system users as a main priority, this outcome seems to have failed (Braathen et al. 2013; Mlenzana & Mji 2010; Vergunst et al. 2015). But, how does rehabilitation fit into such an approach and does rehabilitation qualify as a strategy for addressing some of these social determinants of health?

### Situating rehabilitation

One of the key goals of rehabilitation is participation at all levels of community. This participation improves functionality and contributes to wellness and quality of life. It is proposed that access to rehabilitation services is a good strategy for building an effective health system that addresses social determinants for health and well-being. Rehabilitation should be central to any public health approach and should not remain a privileged service for just a few clients who are in a position to pay for rehabilitation services. Rehabilitation has to move out of the medical model and take its rightful position within the public health arena if it aims at fulfilling its role as proposed in the 1978 strategy of PHC. For rehabilitation to achieve this, it needs to be guided by cultural and contextual issues of the people it renders services to and draw relevant and responsive programmes that will not focus only on a person with already existing impairments but will also cater for everybody, thereby engaging the community in disease prevention and health promotion programmes. This will build healthy empowered communities. Kaseje et al. (2005) consider the notion of community empowerment as the involvement of community members in the design of their own health service provision, acknowledging existing assets which are incorporated into their conceptualisation of health.

Taking a citizen-centred approach to designing a DHS would facilitate the implementation of a responsive PHC system (Fryatti, Hunteri & Matsosoi 2014). Citizen-centred service delivery ensures that clients ‘get what they want and that resources are allocated accordingly’ (Pal 2006:230) by focusing on citizens first and assessing their needs and levels of satisfaction (Baig, Dua & Riefberg 2014). In combination with the Batho Pele principles (Khoza & Du Toit 2011) a citizen-centred approach may nurture accountability of the health system services to its users.

The current re-engineering of PHC could provide extraordinary opportunities for community service professionals, particularly rehabilitation professionals, to extend their scope of practice across the sectors as well as across the different levels of care, especially at a community level with the aim to strengthen the health service. However, the already mentioned barriers do need to receive serious attention and the scope of practice of community service professionals does need to be revised and interpreted within the tenets of the PHC strategy. There is a need to look locally for innovations related to PHC and debate about options of what works and what does not work while keeping a close watch on quality, access, equity and costs (Fryatti et al. 2014). Users of the public sector call for professionals to come to the communities to listen, learn and join hands with the users of health services at community level (Ned 2013). Responding to this call will assist in planning and providing services that are responsive to population needs as well as their contextual and cultural demands and would facilitate communities becoming active participants in the creation of their health and developing solutions for improving their health status.

## Conclusion: Implications for disability

The reality is that, within this health system, persons with disabilities in rural areas continue to struggle with healthcare and rehabilitation. The extricable link between poverty, disability and rurality presents persons with disabilities with many other challenges for community integration. Healthcare and rehabilitation need to prevent disabilities and the worsening of existing disabilities. Free healthcare is not always free for persons with disabilities who live in rural areas as they incur transport (amongst others) expenses and difficulties getting to nearest facilities considering the poor infrastructure, high levels of unemployment and limited access to key services like healthcare and education (Vergunst 2016).

Deconstructing the institutionalisation of services through a process of transversalism (advocating and shifting) is important for rehabilitation professionals to learn about the disabling consequences of the rural environment as experienced by persons with disabilities. Professionals need to understand that daily struggles and barriers faced by persons with disabilities particularly in rural areas constitute the disability. This understanding would allow for reflexivity amongst the professionals and the facilitation of collaboration with persons with disabilities to inform contextually responsive health and rehabilitation services that address disability issues at community level and address the barriers hindering full community integration. Collaboration between communities and the professionals would birth a comprehensive understanding of how to effectively address disability issues in context. It could also interrogate the design of the health system with regard to the different levels of care and availability of human and material resources for such services in all levels. It is worth highlighting that the community should be recognised and used effectively as the primary care level where persons with disabilities are to be successfully integrated. In the process of partnering with communities, this suggested process of rooting and shifting is important so that rehabilitation professionals are careful not to reproduce the same exclusion and marginalisation of persons with disabilities.

Rehabilitation professionals should be conscious of the hegemonies they maintain and the political dynamics (the institutionalisation as an oppressive space), therefore further silencing persons with disabilities and limiting their full integration into their communities. If we do not become conscious and analyse these oppressive forces embedded within the system and within which we are maintaining in the way we practise rehabilitation in rural areas, we will never understand why persons with disabilities either do not come back for follow-ups or why they do not find meaning in these services and why they constantly struggle to be integrated post-discharge from the institutions. More research in this regard is needed. This approach would promote a way of looking at rehabilitation from the user’s perspective, bringing the whole issue of participation and a way of living that arises from the users’ definitions of health. The rehabilitation service should be informed by the communities it serves and align itself with the service needs of these communities through drawing on the knowledge of the community. This would halt the promotion of sick communities and support communities in creating health for themselves. A true participatory sense of a population-centred approach would emerge and a true sense of ‘nothing about us without us’ will hold true.

The social health determinant approach focusing on access to rehabilitation services as a preventative measure will indirectly address the quadruple burden of disease and disability as experienced by the most vulnerable populations in South Africa. Kaseje et al. (2005) proposed a dialogue spiral involving all citizens to contribute to service development. In the same way, persons with disabilities and their communities could be empowered to co-plan appropriate and relevant rehabilitation services and play an active role in maintaining their full integration. Community service professionals could work with health workers from the health sector, development workers from social development, traditional leaders in their different levels and local government to assist communities to assert themselves and participate in activities that facilitate better integration of persons with disabilities in their villages. These other levels of care, such as the home-based carers and community-based workers, could be further explored in terms of how they can work together and complement the community service rehabilitation professionals in providing continuity of care, linking PHC with existing community-based developments while also fostering reciprocal capacity building in the process (Ned 2013). This includes exploring the health system already existing in communities, such as the traditional health system. It is believed that this teamwork approach could further strengthen the DHS and its decentralisation process, thereby fostering meaningful and dignified community participation of persons with disabilities. It would also strengthen the advocacy skills of community service therapists, with improved use of bottom-up approaches to connecting with populations. Sherry (2016) concurs and suggests that the missing layer is empowered and empowering engagement between rural people with disabilities and PHC workers in South Africa. She asserts that rehabilitation professionals also constitute a resource for the broader PHC team, providing both formal and informal training on disability and specific input on adapting health services to take account of disability. Thus, persons with disabilities, when empowered, may also play a role as critical rehabilitation team players as peer supporters and community rehab workers (amongst other players). This teamwork would strengthen the advocacy work and mobilise resources to better facilitate full community integration of persons with disabilities. It is our role as rehabilitation and disability professionals to then facilitate the participation valued by persons with disabilities, participation that is empowering characterised by collaboration and shared understanding and power (Sherry 2016). For this to effectively happen, rehabilitation professionals need to open up to unlearn old ways and relearn from communities they serve. They also need to understand that rehabilitation is a fluid entity that is influenced by both contextual and cultural aspects of the communities they serve. The best indicators for the rehabilitation of a population are those drawn at community level.

## References

[CIT0001] African National Congress (ANC), 1994, *A basic guide to the reconstruction and development programme*, South Africa: A Policy (Framework), Umanyano Publications, Johannesburg.

[CIT0002] Baig A., Dua A. & Riefberg V, 2014, *Putting citizens first: How to improve citizens’ experience and satisfaction with government services*, McKinsey Center for Government, Washington, DC.

[CIT0003] Boneham A. & Sixsmith J.A, 2005, ‘The voices of older women in a disadvantaged community’, *Social Science and Medicine* 62(2), 269–262. https://doi.org/10.1016/j.socscimed.2005.06.003 16039027 10.1016/j.socscimed.2005.06.003

[CIT0004] Braathen S.H., Swartz L., Mannah H., Maclachlan M., Mji G. & Verngust H, 2013, ‘Understanding the local context for the application of global mental health: A rural South African case study’, *International Health* 5(1), 38–42. https://doi.org/10.1093/inthealth/ihs016 24029844 10.1093/inthealth/ihs016

[CIT0005] Cook J.S, 2005, ‘Use of traditional Mi’kmaq medicine amongst patients at First Nations Community Health Center’, *Canadian Journal of Rural Medicine* 10(2), 95–99. 15842792

[CIT0006] County of Los Angeles Public Health, 2013, *Social determinants of Health: How social and economic factors affect Health*, County Department of Public Health, Los Angeles, CA.

[CIT0007] Department of Health (DOH), 1997, *White paper for the transformation of the health system in South Africa*, Government Gazette, #17910, National Department of Health, Pretoria.

[CIT0008] Department of Health (DOH), 2000a, *Pharmacist intern letter*, Public Service, Department of Health, Pretoria.

[CIT0009] Department of Health (DOH), 2000b, *Rehabilitation for all: National rehabilitation policy*, Government Printers, Pretoria.

[CIT0010] Department of Health (DOH), 2002, *A district hospital service package for South Africa*, South African Government, Pretoria.

[CIT0011] Department of Health (DOH), 2007, *Department of health allied health professions community service: 2007 letter*, Department of Health, Pretoria.

[CIT0012] Department of Health (DOH), 2010, *Re-engineering primary health care in South Africa-discussion document*, viewed 04 May 2013, from http://www.foundation.co.za/District-Health-Management/Viewcategory.html

[CIT0013] Department of Health (DOH), 2011a, *Human resources for Health South Africa. HRH strategy for the health sector: 2012/13–2016/17*, South African Government, Pretoria, viewed 03 February 2016, from http://www.gov.za/documents/human-resources-health-south-africa-hrh-strategy-health-sector-201213-201617

[CIT0014] Department of Health (DOH), 2011b, *National Health Insurance in South Africa. Policy (Green) Paper*, South African Government, Pretoria.

[CIT0015] Department of Health (DOH), 2013, *Provincial Government of the Western Cape, South Africa. Health Western Cape. Healthcare 2010: Health Western Cape’s plan for ensuring equal access to quality health care*, viewed 03 February 2016, from http://capegateway.gov.za/eng/pubs/news/2005/apr/103445

[CIT0016] Department of Health-National (DOH), 2015, *Framework and strategy for disability and rehabilitation service in SA*, South African Government, Pretoria.

[CIT0017] Diez Roux A.V, 2012, ‘Conceptual approaches to the study of health disparities’, *Annual Review of Public Health* 33, 41–58. https://doi.org/10.1146/annurev-publhealth-031811-124534 10.1146/annurev-publhealth-031811-124534 PMC3740124 22224879

[CIT0018] Doherty J.E. & Couper I, 2016, ‘Strengthening rural health placements for medical students: Lessons for South Africa from international experience’, *SAMJ* 106(5), 524–527. https://doi.org/10.7196/SAMJ.2016.v106i5.10216 27138676 10.7196/SAMJ.2016.v106i5.10216

[CIT0019] Dookie S. & Singh S, 2012, ‘Primary health services at district level in South Africa: A critique of the primary health care approach’, *BMC Family Practice* 2(13), 67 https://doi.org/10.1186/1471-2296-13-67 10.1186/1471-2296-13-67 PMC3403923 22748078

[CIT0020] Hendricks S.J.H., Buch E., Seekoe E., Bossert T. & Roberts M, 2015, ‘Decentralisation in South Africa: Options for district health authorities in South Africa’, in Padarath A., King J. & English R. (eds.), *South African health review 2014/15*, pp. 59–72, Health Systems Trust, Durban.

[CIT0021] Flint A, 2015, ‘Traditional healing, biomedicine and the treatment of HIV and AIDS: Contrasting South African and Native American experiences’, *International Journal of Environmental Research and Public Health* 12(4), 4321–4339. https://doi.org/10.3390/ijerph120404321 25903057 10.3390/ijerph120404321 PMC4410250

[CIT0022] Flint A. & Payne J, 2013, ‘Reconciling the irreconcilable? HIV and AIDS and the potential for middle ground between the traditional and biomedical healthcare sectors in South Africa’, *Forum for Development Studies* 40(1), 47–68. https://doi.org/10.1080/08039410.2012.702681

[CIT0023] Frenk J., Chen L., Bhutta Z.A., Cohen J., Crisp N., Evans T. et al., 2010, ‘Health Professionals for a new century: Transforming education to strengthen health systems in an interdependent world’, *The Lancet* 376(9756), 1923–1958. https://doi.org/10.1016/S0140-6736(10)61854-5 10.1016/S0140-6736(10)61854-5 21112623

[CIT0024] Fryatti R., Hunteri J. & Matsosoi P, 2014, ‘Innovations in primary health care: Considerations for National Health Insurance’, in Padarath A., King J. & English R. (eds.), *South African health review* 2013–2014, pp. 33–44, Health Systems Trust, Durban.

[CIT0025] Gaede B. & Versteeg M, 2011, ‘The state of the right to health in rural South Africa’, in Padarath A. & English R. (eds.), *South African health review 2011*, Health Systems Trust, Durban, viewed 03 February 2016, from http://www.hst.org.za/publications/south-african-health-review-2011

[CIT0026] Gessler M.C., Msuya M.H.H. & Nkunya A, 1995, ‘Traditional healers in Tanzania: Sociocultural profile and three short portraits’, *Journal of Ethnopharmacology* 48, 145–160. https://doi.org/10.1016/0378-8741(95)01295-O 8719975 10.1016/0378-8741(95)01295-o

[CIT0027] Hatcher A.A., Onah M., Kornik S., Peacocke J. & Reid S, 2014, ‘Placement, support, and retention of health professionals: National, cross-sectional findings from medical and dental community service officers in South Africa’, *Human Resources for Health* 12, 14, viewed 03 February 2016, from http://human-resources-health.com/content/12/1/2014 24571826 10.1186/1478-4491-12-14 PMC3975958

[CIT0028] Hess-April L, 2013, ‘Occupational therapy graduates’ conceptualisation of occupational justice in community service practice in South Africa: A UWC case study’, doctoral thesis, University of Western Cape.

[CIT0029] Kaseje D.C.O., Juma P.A. & Missie Oindo M.P.A, 2005, ‘Public health in Africa: What is new – The context, the gains, the losses, the renewed public health, and the way forward?’, *Kidney International Supplements* 68(98), S49–S59. https://doi.org/10.1111/j.1523-1755.2005.09810.x 10.1111/j.1523-1755.2005.09810.x 16108972

[CIT0030] Khan N.B., Knight S. & Esterhuizen T, 2009, ‘Perceptions of and attitudes to the compulsory community service programme for therapists in KwaZulu-Natal’, *South African Journal of Communication Disorders* 56, 17–22. 20235490

[CIT0031] Khoza V.L. & Du Toit H.S, 2011, ‘The Batho Pele principles in the health services’, *Professional Nursing Today* 15(3), 8–11.

[CIT0032] Magnussen L., Ehiri J. & Jolly P, 2004, ‘Comprehensive versus selective primary health care: Lessons for global health policy’, *Health Affairs* 23(3), 167–176. https://doi.org/10.1377/hlthaff.23.3.167 10.1377/hlthaff.23.3.167 15160814

[CIT0033] Mash B, 2004, ‘Perceptions of the role of the clinical nurse practitioner in the Cape Metropolitan doctor-driven community health centres’, *South African Family Practice* 46(10), 21–25. https://doi.org/10.1080/20786204.2004.10873138

[CIT0034] Matsoso M.P. & Strachan B, 2011, ‘Human resources for health for South Africa: HRH strategy for the health sector 2012/13-2016/17’, in Padarath A. & English R. (eds.), *South African health review*, Health Systems Trust, Durban, viewed 03 February 2016, from http://www.hst.org.za/publications/south-african-health-review-2011

[CIT0035] Mayosi B.M., Lawn J.E., Van Niekerk A., Bradshaw D., Abdool Karim S.S. & Coovadia H.M, 2012, ‘Health in South Africa: Changes and challenges since 2009’, *The Lancet* 380(9858), 2029–2043. https://doi.org/10.1016/S0140-6736(12)61814-5 10.1016/S0140-6736(12)61814-5 23201214

[CIT0036] Mji G, 2012, ‘The health knowledge utilised by rural older Xhosa women in the management of health problems in their home situation, with a special focus on indigenous knowledge’, doctoral thesis, University of Stellenbosch.

[CIT0037] Mlenzana N. & Mji G, 2010, ‘The management of minor health ailments by doctors, clinical nurse practitioners and clients at primary level of care in Cape Town’, *Journal of Community Health Sciences* 5(2), 37–44.

[CIT0038] Morgan A. & Ziglio E, 2007, ‘Revitalising the evidence base for public health: An assets model’, *Promotion and Education Supplement* 15(2), 17–22. 10.1177/10253823070140020701x 17685075

[CIT0039] Moshabela M., Zuma T. & Gaede B, 2016, ‘Bridging the gap between biomedical and traditional health practitioners in South Africa’, in *South African health review*, Health Systems Trust, Durban, viewed 05 November 2016, from http://www.hst.org.za/sites/default/files/SAHR_2016.pdf

[CIT0040] Mostert-Wentzel K., Frantz J. & Van Rooijen A.J, 2013, ‘A model for community physiotherapy from the perspective of newly graduated physiotherapists as a guide to curriculum revision’, *African Journal of Health Professions Education* 5(1), 19–25. https://doi.org/10.7196/AJHPE.203

[CIT0041] Mpofu R, 1995, ‘Community-based rehabilitation: A way forward in Africa’, *SAJPA* 51(3), 39–41.

[CIT0042] Naledi T., Barron P. & Schneider H, 2011, ‘Primary health care in SA since 1994 and implications of the new vision for PHC Re-engineering’, in Padarath A. & English R. (eds.), *South African health review*, Health Systems Trust, Durban, viewed 03 February 2016, from http://www.hst.org.za/publications/south-african-health-review-2011

[CIT0043] Ned L, 2013, ‘A study to explore the capacity of family and service providers to facilitate participation of disabled youth in accessing opportunities in skills development and employment in Cofimvaba, Eastern Cape’, master’s thesis, University of Cape Town.

[CIT0044] Ng S.T., Lingard L., Hibbert L., Regan S., Phelan S., Stooke R. et al., 2015, ‘Supporting children with disabilities at school: Implications for the advocate role in professional practice and education’, *Disability and Rehabilitation* 37(24), 2282–2290. https://doi.org/10.3109/09638288.2015.1021021 25738906 10.3109/09638288.2015.1021021 PMC4673542

[CIT0045] Pal L, 2006, *Beyond policy analysis: Public issue management in turbulent times*, 3rd edn, Nelson Education, Toronto, ON.

[CIT0046] Paterson M., Green M. & Maunder E.M.W, 2007, ‘Running before we walk: How can we maximise the benefits from community service dietitians in KwaZulu-Natal, South Africa?’, *Health Policy* 82(3), 288–301. https://doi.org/10.1016/j.healthpol.2006.09.013 17101189 10.1016/j.healthpol.2006.09.013

[CIT0047] Penn C., Mupawose A. & Stein J, 2009, ‘From pillars to posts: Some reflections on community service six years on’, *South African Journal of Communication Disorders* 56(1), 8–16. 20235489

[CIT0048] Pillay Y. & Barron P, 2011, ‘The implementation of PHC re-engineering in South Africa’, PHASA Newsletter, 15 November 2011, viewed 08 February 2012, http://www.phasaimplementation-of-phc-re-engineering-in-south-africa.html

[CIT0049] Ramklass S.S, 2009, ‘An investigation into the alignment of a South African physiotherapy curriculum and the expectations of the healthcare system’, *Physiotherapy* 95(3), 216–223. https://doi.org/10.1111/j.1365-2524.2009.00869.x 19635342 10.1016/j.physio.2009.02.004

[CIT0050] Reid S.J, 2001, ‘Compulsory community service for doctors in South Africa – An evaluation of the first year’, *South African Medical Journal* 91, 329–336. 11402906

[CIT0051] Reid S.J. & Conco D, 1999, ‘Monitoring the implementation of community service’, in Crisp N. & Ntuli A. (eds.), *South African health review*, pp. 233–248, Health Systems Trust, Durban.

[CIT0052] Republic of South Africa, 2005, *National Health Act 61 of 2003*, Government Printer, Pretoria.

[CIT0053] Rispel L, 2016, ‘Analysing the progress and fault lines of health sector transformation in South Africa’, in *South African health review*, pp. 17–24, Health Systems Trust, Durban, viewed 05 November 2016, from http://www.hst.org.za/sites/default/files/SAHR_2016.pdf

[CIT0054] Sherry K, 2015, ‘Disability and rehabilitation: Essential considerations for equitable, accessible and poverty-reducing health care in South Africa’, in *South African health review 2014/2015*, pp. 89–100, Health Systems Trust, Durban.

[CIT0055] Sherry K, 2016, ‘Occupations of citizenship: The missing layer in empowered engagement between rural people with disabilities and primary healthcare workers in South Africa’, doctoral thesis, University of Cape Town.

[CIT0056] Thomas B, 2014, ‘Health and health care disparities: The effect of social and environmental factors on individual and population health’, *International Journal of Environmental Research Public Health* 11, 7492–7507. https://doi.org/10.3390/ijerph110707492 25050656 10.3390/ijerph110707492 PMC4113890

[CIT0057] United Nations A/HRC/23/36, General Assembly, Distr.: General 11 March 2013, *Human Rights Council Twenty-third session Agenda item 3 Promotion and protection of all human rights, civil, political, economic, social and cultural rights, including the right to development*, Report of the Special Rapporteur on extreme poverty and human rights, Magdalena Sepúlveda Carmona, United Nations.

[CIT0058] Vergunst R, 2016, ‘Access to health care for persons with disabilities in rural Madwaleni, Eastern Cape, South Africa’, doctoral thesis, University of Stellenbosch.

[CIT0059] Vergunst R., Swartz L., Mji G., MacLachlan M. & Mannan H, 2015, ‘“You must carry your wheelchair” – Barriers to accessing healthcare in a South African rural area’, *Glob Health Action* 8, 29003 https://doi.org/10.3402/gha.v8.29003 10.3402/gha.v8.29003 PMC4592846 26434691

[CIT0060] Versteeg M., Du Toit L. & Couper I, 2013, ‘Building consensus on key priorities for rural health care in South Africa using the Delphi technique’, *Global Health Action* 6(19522), 119–126. https://doi.org/10.3402/gha.v6i0.19522 10.3402/gha.v6i0.19522 PMC3556680 23364081

[CIT0061] Weeramanthri T.S. & Bailie R.S, 2015, ‘Grand challenges in public health policy’, *Frontiers Public Health* 3, 29 https://doi.org/10.3389/fpubh.2015.00029 10.3389/fpubh.2015.00029 PMC4332160 25763366

[CIT0062] Wentzel K., Frantz K. & Van Rooijen A.J, 2013, ‘A model for community service physiotherapy from the perspective of newly graduated physiotherapists as a guide to curriculum revision’, *African Journal of Health Professions Education* 5(1), 19–25.

[CIT0063] Werner D. & Sanders D 1997, ‘The demise of primary health care and the rise of the child survival revolution’, in Werner. D,, Sanders D,., Weston J,., Babb S,. & Rodriguez B., *Questioning the solution: The politics of primary health care and child survival*, pp. 23–30, Health Wrights, Palo Alto, CA.

[CIT0064] World Health Organization (WHO), 2007, *Community health workers: What do we know about them? The state of the evidence on programmes, activities, costs and impact on health outcomes of using community health workers, Evidence and Information for Policy*, Department of Human Resources for Health, Geneva A report by Uta Lehmann and David Sanders School of Public Health University of the Western Cape.

[CIT0065] World Health Organization (WHO), 2010a, *Increasing access to health workers in remote and rural areas through improved retention: Global policy recommendations*, WHO, Geneva. 23741785

[CIT0066] World Health Organization (WHO), 2010b, *Monitoring the building blocks of health systems: A handbook of indicators and their measurement strategies*, WHO, Geneva.

[CIT0067] Wreford J, 2005, *Negotiating relationships between biomedicine and Sangoma: Fundamental misunderstandings, avoidable mistakes*, Centre for Social Science Research, University of Cape Town, Cape Town.

[CIT0068] Yin R.K, 2014, *Case study research: Design and methods*, 5th edn, Sage, Thousand Oaks, CA.

[CIT0069] Zonke M, 2005, ‘The exploration of indigenous health knowledge carried by the older persons in the care of clients presenting with minor health ailments’, master’s thesis, University of Cape Town, Cape Town, South Africa.

[CIT0070] Zühlke L.J. & Engel M, 2013, ‘The importance of awareness and education in prevention and control of RHD’, *Global Heart* 8(3), 235–239. 25690501 10.1016/j.gheart.2013.08.009

